# Advances in Lipid Extraction Methods—A Review

**DOI:** 10.3390/ijms222413643

**Published:** 2021-12-20

**Authors:** Ramesh Kumar Saini, Parchuri Prasad, Xiaomin Shang, Young-Soo Keum

**Affiliations:** 1 Department of Crop Science, Konkuk University, Seoul 143-701, Korea; saini1997@konkuk.ac.kr; 2 Institute of Biological Chemistry, Washington State University, Pullman, WA 99164, USA; prasad.parchuri@wsu.edu; 3 Jilin Provincial Key Laboratory of Nutrition and Functional Food, Jilin University, Changchun 130062, China; xmshang@jlu.edu.cn

**Keywords:** lipidomics, Folch method, Bligh and Dyer method, Soxhlet extraction, supercritical CO_2_ extraction, pre-treatments, green solvents

## Abstract

Extraction of lipids from biological tissues is a crucial step in lipid analysis. The selection of appropriate solvent is the most critical factor in the efficient extraction of lipids. A mixture of polar (to disrupt the protein-lipid complexes) and nonpolar (to dissolve the neutral lipids) solvents are precisely selected to extract lipids efficiently. In addition, the disintegration of complex and rigid cell-wall of plants, fungi, and microalgal cells by various mechanical, chemical, and enzymatic treatments facilitate the solvent penetration and extraction of lipids. This review discusses the chloroform/methanol-based classical lipid extraction methods and modern modifications of these methods in terms of using healthy and environmentally safe solvents and rapid single-step extraction. At the same time, some adaptations were made to recover the specific lipids. In addition, the high throughput lipid extraction methodologies used for liquid chromatography-mass spectrometry (LC-MS)-based plant and animal lipidomics were discussed. The advantages and disadvantages of various pretreatments and extraction methods were also illustrated. Moreover, the emerging green solvents-based lipid extraction method, including supercritical CO_2_ extraction (SCE), is also discussed.

## 1. Introduction

Lipids are essential biomolecules responsible for mediating various physicochemical properties of the membrane and modulating vital cellular functions such as subcellular compartmentalization, trafficking, signaling, and regulation of membrane and non-membrane proteins [1]. The International Lipid Classification and Nomenclature Committee (ILCNC) classified lipids into eight categories—namely, (1) fatty acids, (2) glycerolipids (e.g., triacylglycerols, TAGs), (3) glycerophospholipids (GPLs; fatty acid-glycerol-phosphate ester), (4) sphingolipids, (5) sterol lipids, (6) prenol lipids, (7) saccharolipids, and (8) polyketides [2]. Among these, GPLs, commonly known as phospholipids (PLs), TAGs, and sterols (mainly cholesterol in animals), are the most common type of fat found in plants and animals [3]. Moreover, in microalgae, thraustochytrids, fish, krill, and plant seeds, the nutritionally important omega-3 polyunsaturated fatty acids (PUFAs) are generally attached to TAGs [4,5].

The complete profile of lipid species present in a tissue, organelle or cell, refers to the lipidome, whereas the study of lipid profiles within biological systems can be called lipidomics [6]. The modern multi-dimensional liquid chromatography (LC)- mass spectrometry (MS)-based lipidomics enables us to accurately identify lipid alterations (metabolic differences) within individual lipid classes, subclasses, and molecular species [6]. Identifying the lipid alterations can provide vital information related to cellular homeostasis and disease pathogenesis [1].

In lipidomics and biodiesel and vegetable oil production, lipid extraction from the biological tissues is the most crucial step. The chemical and structural diversity of lipids makes efficient extraction using a single experimental approach quite challenging. Moreover, the cellular complexity of biological samples, such as body fluids, tissues, and cells, often requires optimizing extraction techniques. For the extraction of lipids, there are two significant challenges to overcome: extraction efficiency and complete removal of non-lipid contents. The throughput and analyses reproducibility can be substantially enhanced by optimizing the sample preparation methodology precisely.

The selection of appropriate solvent is the most critical factor in the efficient extraction of lipids. The neutral lipids solubilize well in nonpolar organic solvents, but the polar lipids, especially the GPLs, dissolve well in polar solvents. Thus, to efficiently extract lipids from biological tissue, a solvent mixture, including polar to disintegrate the lipids from cell membranes and lipoproteins and nonpolar to dissolve the neutral lipids, is desirable. This concept was first established by Folch et al. [7], who developed an extraction method using a 2:1 (*v*/*v*) solvent mixture of chloroform/methanol, followed by purification of the extracts with a salt solution (0.003 N CaCl_2_ or MgCl_2_, or 0.05 N NaCl or KCl). Bligh and Dyer [8] modified the existing method of Folch et al. [7] and obtained a rapid method for total lipid extraction from animal tissues.

In addition to selecting appropriate solvents, the disintegration of complex and rigid cell-wall of plants, fungi, and microalgal cells facilitates the solvent penetration and extraction of lipids [9,10]. This is achieved by various mechanical, chemical, and physicochemical, and enzymatic treatments (called pretreatments) before the solvent extraction [11].

In the six decades since introducing the Folch Method [7] and Bligh and Dyer method [8], many investigators have applied the method with various adaptations. Most adoptions were made for the rapid one-step extraction, which facilitates the high throughput screening [12,13,14]; some studies proposed the substitution of toxic chloroform [15,16], while some adaptations were made to recover the specific lipids [17].

This review discusses the classical Folch Method [7] and Bligh and Dyer method [8] and modern modifications of these methods regarding the use of health and environmentally safer solvents and rapid single-step extraction. At the same time, some adaptations were made to recover the specific lipids. Moreover, the high throughput lipid extraction methodologies used for liquid chromatography-mass spectrometry (LC-MS)-based plant and animal lipidomics were discussed. In addition, the disintegration of complex and rigid cell-wall of plants, fungi, and microalgal cells by various mechanical, chemical, and physicochemical, and enzymatic treatments facilitate the solvent penetration and extraction of lipids. The advantages and disadvantages of various pretreatments and extraction methods were also illustrated. Moreover, the emerging green solvents-based lipid extraction method, including supercritical CO_2_ extraction (SCE), is also discussed.

## 2. Pretreatments before Extraction

It is essential to disintegrate the cells before lipid extraction to improve the mass transfer to the extraction solvent. Several physical, mechanical, chemical, and biological (enzymatic) pretreatments are utilized to disrupt the rigid cell wall of oleaginous biomass [9,18,19]. Specifically, the complex and rigid cell wall of microalgae hinders solvent penetration, resulting in low extraction of lipids [9]. Thus, the lipid-extraction from microalgae includes cell-wall disruption by appropriate pretreatments followed by lipid extraction by solvent(s) [9]. Moreover, with the pretreatments, a satisfactory yield of lipids can be obtained from the wet algal biomass, thus eliminating the costly dehydration process [20].

The mechanical and physical pretreatment methods are expeller press, bead milling, ultrasonication, microwave, high-speed and high-pressure homogenizer, laser, hydrodynamic cavitation, autoclaving, and pulsed electric field in use [9,18,21,22,23,24]. The selection of these methods primarily depends on the moisture contents of the sample and microalgal species (distinct cell-wall characteristics) [9,10,25,26,27].

Byreddy et al. [25] compared the efficiency of nine organic solvents and solvent combinations with six different cell disruption methods to extract the lipid from *Schizochytrium* and *Thraustochytrium* sp. (natural and commercially exploited sources of long-chain polyunsaturated fatty acids), including bead vortexing, grinding with liquid nitrogen, water bath, osmotic shock, sonication, and shake mill. Among the solvents, chloroform/methanol (2:1, *v*/*v*) showed the highest yield, followed by chloroform/n-hexane (2:1, *v*/*v*). Among the cell disruption methods, the highest lipids yield was obtained using osmotic shock, which was 2.8-fold higher than control. Interestingly, grinding produced the second-highest yield of lipids from *Schizochytrium* sp. However, it was not adequate for *Thraustochytrium* sp., which shows the requirements of species-specific pretreatment methods.

In the enzymatic pretreatment methods, cellulolytic enzymes, e.g., amylase, cellulase, papain, pectinase, hemicellulose, β-glucosidases, β-1,3-glucomannanase, and xylanase enzymes are used to disintegrate the cell wall of oleaginous yeast and microalgae [18,28,29,30,31,32]. It has been suggested that a combination of various treatments may provide a high extraction yield of lipids [18].

Furthermore, when extracting lipids from legumes and cereals, the hydration of finely ground samples can substantially help deeper penetration of the solvents, resulting in a substantially higher yield of total lipids [33]. The mode of action, advantages, and disadvantages of various pretreatment methods are summarized in Table 1.

## 3. Selection of Appropriate Extraction Solvent(s)

Extraction of lipids from cells and tissues is primarily a mass transfer operation, either by the direct release of lipids in bulk with disruption of the cells or diffusion of lipids across the cell wall [57]. The polarity of solvent substantially influences the diffusion of lipids across the cell wall, thus substantially influencing the extraction efficiency of lipids [57]. Moreover, lipids are associated with macromolecules such as proteins and polysaccharides [3]. Thus, extraction solvent should have high polarity (high dielectric constants) that can access regions of ion-dipole interactions and hydrogen bonding and can disrupt these interactions. Moreover, the nature of the solvent substantially influences the nature of the lipids contained in the extract [58]. The list of solvents tested for the efficient extraction of lipids plants, animals, and microbes are illustrated in Table 2.

In general, nonpolar solvents efficiently extract the TAGs [59,64], while polar solvents, such as acetonitrile and ethanol, and chloroform provide a high yield of Pls [59]. De Jesus [65] recorded the higher yield of lipids using the Bligh and Dyer method from the wet microalgae compared to the Folch method. The authors suggested the higher yield from Bligh and Dyer method resulted from the higher the polarity in the medium due to the addition of water, which improves the phase separation and lipid yield.

In addition to these factors, a choice of solvent(s) for lipid extraction also depends on several other factors, such as volatility (for easy separation after extraction), freedom from toxic, mutagenic, or reactive impurities (to avoid reaction with the lipids), ability to form an aqueous two-phase system (to remove non-lipids compounds), health and environmental concerns, and price. In recent years, the impact of solvents on the environment is also a critical decisive criterion for selecting solvents [58,59].

The classical methods use a mixture of chloroform/methanol to extract lipids [7,8]. Methanol used in the classical methods does not primarily serve as a lipids extraction solvent as it is miscible (mixes thoroughly) in water. In fact, it disrupts the electrostatic forces or hydrogen bonding networks between proteins and lipids [3]. The chloroform predominantly mediates the actual diffusion and mass transfer of lipids from cells. Moreover, the water-immiscible properties of chloroform help in the formation of a biphasic system. Methanol can be replaced by ethanol or isopropanol (2-propanol or propan-2-ol). Ethanol offers a similar polarity to disruption membrane-lipids-protein as methanol [66]. However, isopropanol may be weaker in disrupting such interactions due to larger hydrophobic moiety.

Ranjan et al. [57] comparatively investigated the extent of microalgal lipid extraction with four major techniques. This study obtained the highest yield of total lipids with chloroform/methanol extraction with sonication, probably due to the combined effects of diffusion of lipids across the cell wall with the direct release of lipids in bulk with disruption, followed by the Bligh and Dyer method. In contrast, the yield was lowest by Soxhlet extraction and sonication with n-hexane. Interestingly, in this study, even with ultrasonication utilized, the micrographs of the algal biomass showed incomplete disruption of microalgal cells. Therefore, the authors suggested that the diffusion mechanism (controlled by the solvent) is the most contributing mechanism of lipid extraction.

Among the different solvents with varied polarity index (PI), such as n-hexane (PI = 0), ethyl acetate (PI = 4.4), acetone (PI = 5.1), n-hexane/acetone (1:1, *v*/*v*; PI = 2.5), ethanol/water (96:4, *v*/*v*; PI = 5.4), and water (PI = 10.2) based solvents tested for the mechanical extractions of oil from unroasted Argan (*Argania spinosa* L.) seeds, the n-hexane-acetone yielded the highest amounts of lipids (39.7%), followed by acetone (36.5%). In contrast, the solvents with low PU were not effective [58].

Moreover, the water content in the food largely influences the extraction yield of lipids [59]. The high contents of the water in the food (e.g., egg yolk and wet microalgae) inhibit the contact between lipids and nonpolar solvent (e.g., n-hexane), resulting in a low yield of lipids [59,65], compared to the food with low content of water (e.g., egg powder, dry microalgal biomass) [59,65]. However, the influence of water can be minimized by using solvents with medium polarity (e.g., ethyl acetate/ethanol).

In a comparison among chloroform/methanol (Folch methods) with acetone, ethanol, ethyl acetate, isopropanol, isohexane (2-methylpentane), n-hexane, and subcritical butane for the extraction of lipids and other lipophilic constituents from krill meal, the chloroform/methanol provided the highest yield of total lipids, followed by ethanol and isopropanol, while acetone resulted in the lowest yield [60]. Interestingly, in this study, carotenoids and sterols were best extracted in acetone, which shows the selectivity of acetone in extracting the polar carotenoids and other minor lipophilic constituents.

Ren et al. [27] recently investigated the effect of four solvent systems comprising acetone, chloroform/methanol, dichloromethane/methanol, and chloroform/methanol/water, with several other parameters to isolate the lipid from microalgae. The chloroform/methanol/water produced the highest lipid yield. Interestingly, in this study, microscopic examinations revealed that adding water to the extraction solvent triggered the destruction of the microalgal cell wall, resulting in an enhanced yield of lipids.

## 4. Lipid Extraction Methods

Despite the availability of appropriate methodology of one-step extraction and methylation of fatty acids [64,67], most studies analyzing the fatty acid composition are based on three distinct steps, (1) extraction of crude lipids and gravimetric analysis of total crude lipids in the sample, (2) saponification and methylation, and (3) analysis by gas chromatography (GC)- flame ionization detector (FID) and GC-MS [68,69,70]. Moreover, using the techniques such as near-infrared spectroscopy, nuclear magnetic resonance, Raman spectroscopy, and hyperspectral imaging, nondestructive determination of fat content and fatty acids composition is also possible [71]. However, the high cost of these instruments and difficulties in assessing the minor amounts of fatty acids, limiting the wide use of these techniques for routine analysis.

The selection of appropriate methods plays a critical role in the efficient extraction of major and minor lipids (qualitatively and quantitatively). The selection of appropriate methods is based on the origin of the sample (plant and animal), physical state (tissue or fluid), moisture contents, and lipid contents [72]. Moreover, the extraction methods can also be based on the subsequent requirement of the extracted lipids.

### 4.1. Classical Methods: Bligh and Dyer and Folch Methods

The Folch method [7] and Bligh and Dyer method [8], published in 1957 and 1959, respectively, are considered gold standards for the extraction of lipids [73]. Though these methods were originally developed to extract lipids from animal tissues, the high efficiency of the chloroform/methanol in extracting major lipid classes, these methods are widely followed to extract lipids from a wide range of plants and animals (fluids and tissues) samples. The Folch method is generally preferred to extract lipids from solid tissue, whereas the Bligh and Dyer method is considered advantageous for biological fluids [3].

**Figure 1:**
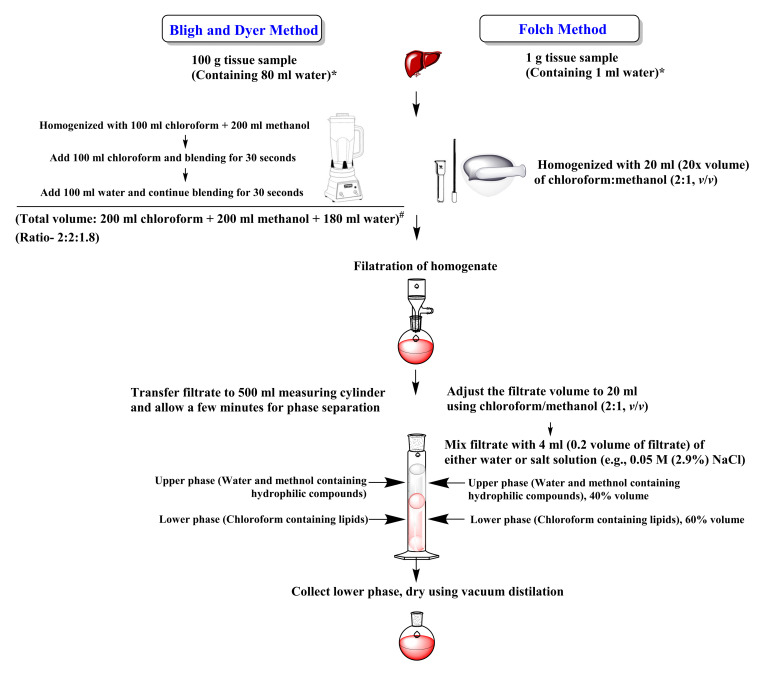
Comparison of Bligh and Dyer method [8] and Folch method [7] of lipid extraction. * Assumption of 100% or 80% water in the sample. # For quantitative analysis, re-extraction of residues with 100 mL chloroform and rinsing with 50 mL chloroform is recommended.

An outline of these methods is illustrated in Figure 1. The main differences between the protocols of Folch et al. [7] and Bligh and Dyer [8] are the ratio of chloroform/methanol/water (2:1:0.75 in Folch and 1:1:0.9% in Bligh and Dyer method), the volume of the solvent system (20 times of sample in Folch and four times Bligh and Dyer), assumption of amount of water in the sample (100% in Folch and 80% Bligh and Dyer), and the presence (Folch method) or absence (Bligh and Dyer method), of salts in the added water fraction (Figure 1).

These methods are equally efficient in extracting the total lipids from marine tissue containing <2% lipids [72]. However, for samples containing >2% lipid, Folch methods produced a substantially higher amount of lipids, probably due to the higher proportions of solvent used (20 times of sample), compared to Bligh and Dyer (4 times of sample). Thus, the sample to solvent ratio is a critical factor that influences lipid yield. Ulmer et al. [74] also investigated the extraction of lipids for untargeted lipidomics study and suggested that Folch and Bligh and Dyer method should be employed using a 1:20 (*v*/*v*) sample-to-solvent ratio to obtain the highest yield.

### 4.2. Modified Bligh and Dyer and Folch Methods

The classical Bligh and Dyer and Folch Methods use toxic chloroform/methanol; thus, most modifications were proposed to replace these solvents with comparatively safer or green solvents. In 1978, Hara and Radin [16] proposed n-hexane/isopropanol (3:2 *v*/*v*) to extract lipids from rat or mouse brain tissues. Later, Smedes [15] proposed the use of isopropanol/cyclohexane/water (8:10:11 *v*/*v*/*v*) mixture for the efficient extraction of lipids from marine tissues (plaice, mussel, and herring). Manirakiza [75] compared the Smedes [15] and Bligh and Dyer extraction methods and found that both methods can provide a similar yield of lipids from milk and eggs. While, compared to the Bligh and Dyer method, Smedes [15] methods resulted in a lower yield of lipids from the human serum, probably due to the high proportions of polar PLs (isopropanol is less polar solvating properties than methanol).

The Bligh and Dyer and Folch Methods are multistep (laborious and time-consuming) methods, thus limiting their applications for screening large numbers of samples. Axelsson and Gentili [76] developed a faster single-step procedure for the extraction of total lipids from green microalgae, utilizing biomass (300 mg of wet microalgal paste or 30 mg in dry weight), dispersion in 10 mL solvent system (chloroform/methanol, 2:1, *v*/*v*), followed by addition of 0.73% NaCl water to produce a 2:1:0.8 system of chloroform/methanol/water (*v*/*v*/*v*).

Acidification of extraction medium helps disrupt ionic interactions of charged, polar lipids (e.g., GPLs) with macromolecules, which were not possible by just polar solvents. Retra et al. [17] suggested that a minor modification of the Bligh and Dyer method by adding 0.5% 6M HCl to the second chloroform wash can increase the recovery of acidic PLs from the rat liver and the parasitic helminth *Schistosoma mansoni*. In this method, the first extraction at the natural pH, followed by an acidic extraction, helps extract acidic phospholipids and acid-labile plasmalogens. However, ester bonds are vulnerable to hydrolysis under long exposures to a concentrated acid at elevated temperatures. Thus, in acidification of extraction procedures, the sample should be analyzed immediately, and care should be taken to minimize the hydrolysis by maintaining the pH (2–4) and temperature [3].

### 4.3. Soxhlet Extraction of Lipids

Soxhlet extraction provides a high yield of lipids; however, some studies have reported contrasting results [61,77]. Soxhlet extraction is mostly not suitable for samples containing a high amount of water [75]. In the Soxhlet extraction, diffusion is the only mechanism of diffusion of lipids across the cell wall (not by the direct release of lipids in bulk with disruption of the cell) [57].

In the Soxhlet extraction of lipids, the selection of solvent plays a critical role [78]. Ramluckan et al. [78] investigated the comparative efficiency of thirteen solvents and solvent combinations spanning a range of polarities (0.1 (petroleum ether and n-hexane) to 5.2 (ethanol)) for the extraction of microalgal lipids by the Soxhlet method. In results, ethanol, chloroform, and n-hexane yielded the highest amount of lipids, while acetone was the least effective. Among the binary solvents, chloroform/n-hexane, ethanol/n-hexane, and chloroform/ethanol were investigated in 1:1, 1:2, 1:3, and 3:1 ratio, the highest yield of lipids was obtained with chloroform/ethanol (1:1, *v*/*v*).

Soxhlet extraction provides a high yield of lipids [79]. However, continuous heating at the boiling temperature could lead to lipid oxidation and degradation of heat liable compounds [80]. From seed spices (coriander, caraway, anise, nutmeg), Soxhlet extraction with n-hexane and Folch method yielded a similar amount of total lipids [79]; however, the lipids extracted with Folch method showed higher antioxidant activity, compared to Soxhlet extraction, probably due to the higher extraction of phenolic compounds with Folch method. In contrast, from lentils, the Folch method yielded the highest amount of total lipids (with hydration, 2.47%; without hydration, 1.89), followed by Soxhlet (n-hexane/acetone and n-hexane/methylene chloride), and solid-liquid extraction after hydration with n-hexane-isopropanol and n-hexane-acetone [33]. Similarly, under the optimized condition of n-hexane/isopropanol (3:2; *v*/*v*) extraction assisted with 69.5 min ultrasound treatment at 55 °C and solvent-to sample proportion of 9.12:1 (% *v*/*w*) provided the higher recovery of canola oil compared to Soxhlet extraction [77].

### 4.4. Supercritical CO_2_ Extraction (SCE)

Supercritical CO_2_ extraction (SCE) of lipids involves separating lipids from the biological matrix utilizing the supercritical CO_2_ (green solvent) as the extracting solvent. As properties of CO_2_ can be altered by varying the pressure and temperature, SCE offers selective extractions of metabolites, including lipids. Moreover, the extraction yield can be increased by adding co-solvent (ethanol) [81]. The optimized parameters of supercritical CO_2_ extraction of lipids are illustrated in Table 3.

### 4.5. Extractions of Lipids for Lipidomics Studies

**Figure 2:**
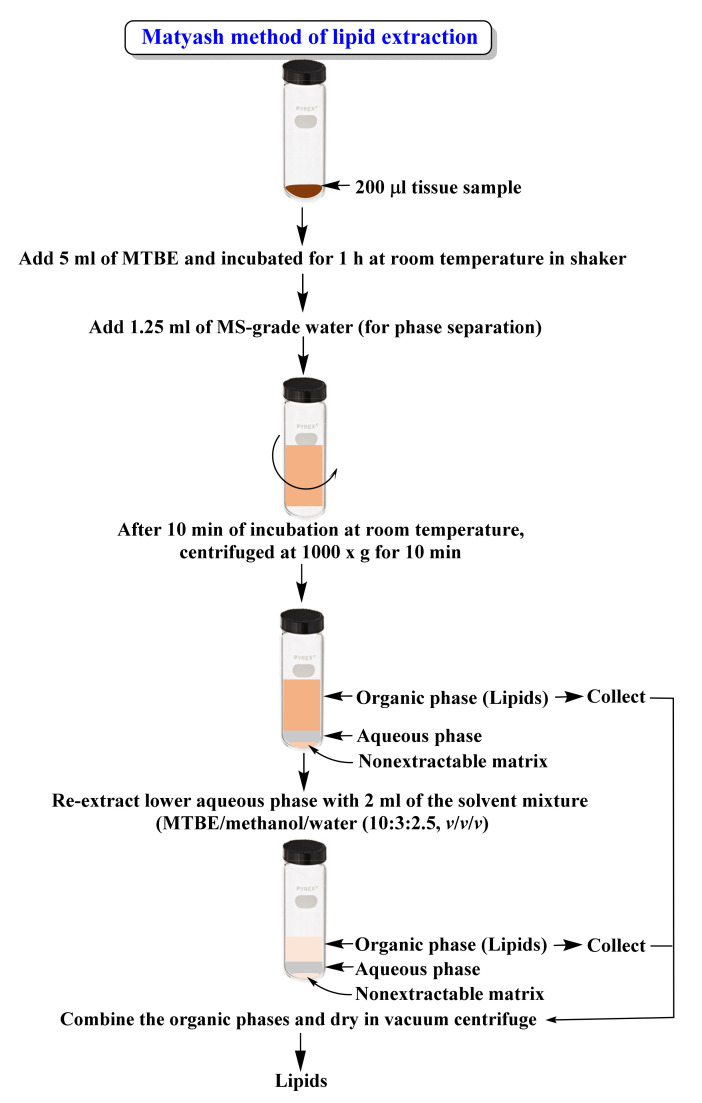
Illustrations showing the Matyash [12] method of lipid extraction.

**Figure 3:**
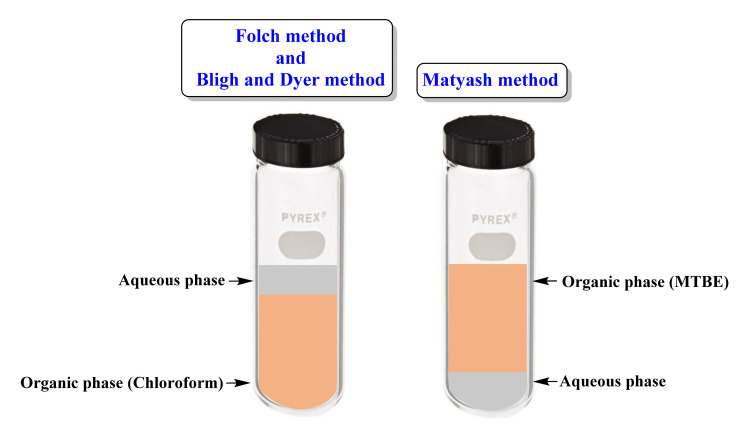
Illustrations showing the phase separation in chloroform-based (Folch method and Bligh and Dyer methods) [7,8] and methyl-tert-butyl ether (MTBE; Matyash Method) [12] based extraction methods. Compared to the lipid-rich chloroform layer below the aqueous phase (in chloroform-based extraction). Moreover, the nonextractable matrix, including proteins (forms at the bottom of the tube), can be removed by centrifugation easily.

In the past decade, advances in LC-MS-based technologies have led to the rapid use of targeted or untargeted lipidomic approaches to understand the physiological and biological roles of lipids in living organisms. However, efficient extraction of structurally diverse lipid species from different samples imposes a bottleneck in lipidomic research. Untargeted lipidomics require the use of non-selectively extraction protocols that can extract all classes of detectable lipids in a sample irrespective of their concentration with minimal contamination of non-lipid molecules such as proteins and carbohydrates. Chloroform/methanol (Folch/Bligh and Dyer) [7,8] or methyl-tert-butyl ether (MTBE) [12] (Figure 2) or butanol/methanol [87] based liquid-liquid extraction protocols are primarily followed for untargeted lipidomics studies in animals. The use of MTBE has the advantages of the lipid-rich organic layer above the aqueous phase, compared to the lipid-rich chloroform layer below the aqueous phase in chloroform/methanol-based extraction (Figure 3). The nonextractable matrix, including proteins (forms at the bottom of the tube), can be removed by centrifugation easily. However, the high volatility of MTBE is a concern and affects the reproducibility of the extraction [88]. Recently, single-phase extraction methods using single organic solvents such as methanol [89], isopropanol [90], or a combination of organic solvents such as butanol/methanol [13] for lipid solubilization and non-lipids precipitation were developed for untargeted lipidomics. As these methods doesn’t involve biphasic solvent separation, they are more convenient, reproducible, and offer an excellent lipid recovery rate over the traditional liquid-liquid extraction protocols. Reis et al. [91] investigated the comparative efficiency of five different solvent extraction protocols: Folch, Bligh and Dyer, acidified Bligh and Dyer, methanol/MTBE, and n-hexane/isopropanol, for the extraction lipids from human LDL, for the lipidomics. In results, the Folch method and acidified Bligh and Dyer method showed the higher yield of total lipid, and overall, these methods were most suitable for broad-based lipidomic studies. In comparison, n-hexane/isopropanol yielded the lowest amount of lipids. Moreover, it was suggested that methanol/MTBE could be used for the sphingolipidomic (lactosyl ceramides and sphingomyelins) studies. Similarly, n-hexane-isopropanol was advised for the non-polar lipids (free fatty acids and cholesterol esters).

Targeted lipidomics mainly focuses on the analysis of specific lipid classes. The chemical structure and polarity of the lipid species of interest drive the selection of the extraction method. Liquid-liquid extraction (LLE) methods using non-polar solvents such as n-hexane or toluene are commonly used to extract highly hydrophobic lipid molecules such as TAGs, diacylglycerols (DAGs), esters of fatty acid, and cholesterol [92]. Chloroform/methanol-based or MTBE-based LLE extraction methods are generally used for intermediate and highly polar lipids such as GPLs and sphingolipids. However, optimizing various parameters such as solvent mixtures and their ratios and acidic /basic extraction conditions are required to avoid recovery differences across lipid classes. For example, the use of mild acidic conditions during LLE improves the recovery of phospholipids such as phosphatidic acid, phosphatidylserine, and phosphatidylinositol [93,94]. Although the above methods can efficiently recovery lipid species of interest, lipid oxidation due to water contamination is a major concern [95]. Meikle et al. [96] proposed rapid preparations of lipid from plasma sample using chloroform/methanol (2:1, *v*/*v*) extraction, followed by separation of supernatant after centrifugation (no partitioning with water required), drying of supernatant under a stream of nitrogen, and resuspension in the desired solvent for LC-MS. Alshehry et al. [13] suggested a single-phase lipid extraction of lipids from human plasma using butanol/methanol (1:1 *v*/*v*) that does not require removing the solvent and reconstitution before LC-MS analysis. Moreover, this method showed a similar yield of plasma lipids with chloroform/methanol (2:1, *v*/*v*). Alternatively, solid-phase extraction (SPE) methods that can be modified based on the source material and do not require solvent/water partitions can be adopted for targeted lipidomics [97].

Lipid extraction methods used in plant lipidomics are majorly derived from Bligh and dyer method with significant improvements. Lipase-based lipid degradation and lipid oxidation are the major bottlenecks in efficiently extracting lipids from plant tissues. Roche et al. [98] showed that boiling wheat seeds in isopropanol to inactivate lipase before lipid extraction by Bligh and Dyer method improved the yield of seed neutral and phospholipids. Ryu and Wang [99] modified the above protocol to inhibit lipase-based lipid degradation and lipid oxidation in a single step by adding 0.01% butylated hydroxytoluene (BHT) to isopropanol and extraction solvents. Welti et al. [14] introduced additional steps to Ryu and Wang [99] method to remove non-lipid molecules, and the modified protocol has been extensively used in plant lipidomics over the past decade. However, this method is time-consuming and labor-intensive. Vu et al. [100] developed a high throughput, streamlined single-step lipid extraction method from the leaf samples. This method involves shaking the leaf samples in a polar solvent mixture for 24 h after quenching with hot isopropanol, and the extracts were directly used for lipidomic analysis. Shiva et al., [101] modified the single-step extraction protocol developed by Vu et al. [100] by shaking the leaf tissues in a solvent mixture of chloroform/isopropanol/methanol/water (30:25:41.5:3.5) with 0.01% BHT. This method is proven to be highly efficient in extracting phospholipids from Arabidopsis and Sorghum leaves. Given the structural diversity and relative hydrophobicity of lipid species, the above methods are not suitable for the extraction of amphiphilic sphingolipids such as glycoinositolphosphorylceramides (GIPCs) from plant tissues [102]. Markham et al. [102] developed a protocol to efficiently extract sphingolipids from plant tissues using isopropanol/n-hexane/water, which is widely followed to analyze plant sphingolipids.

### 4.6. Solid-Phase Extraction (SPE)

SPE is a valuable technique for the isolation and purification of selected lipids along with the enrichment of minor lipid classes. This is generally performed using small cartridges (columns) packed with reversed, normal, or ion exchange sorbents. These cartridges selectively hold the desirable fractions through polar (normal phase), hydrophobic (reverse phase), or ionic interactions while undesirable compounds pass through. The recent studies of SPE of lipids are summarized in Table 4.

### 4.7. Lipid Extraction Utilizing Green Solvents

The conventional technologies of oil recovery from plant seeds (e.g., oilseeds for vegetable oil extraction) use solvent extraction, most commonly with n-hexane for its attributes such as nonpolar nature, low latent heat of vaporization (330 kJ/kg), which facilitates the easy recovery after the extraction, and high solubility of oil [31]. However, using n-hexane as a solvent has led to several consequences such as toxicity, air pollution, and harmfulness that prompted looking for alternative options.

The chloroform/methanol-based solvent used in the traditional Folch and Bligh and Dyer methods efficiently extracts lipids from plants, animals, and microbes. However, researchers have tested other safer solvents due to health and environmental concerns. Among the green solvents, supercritical CO_2_, plant-derived terpenes (e.g., D-limonene, p-cymene, and α-pinene), ionic liquids (non-aqueous salt solution) are emerging [31]. With the help of Conductor-like Screening Model for realistic Solvatation (COSMO-RS), Breil et al. [73] selected ethanol and ethyl acetate as potential substitution of methanol and chloroform for the extraction of lipids from yeast (*Yarrowia lipolytica* IFP29). Moreover, ethanol, ethyl acetate, isopropanol, and n-propanol are good alternatives as they are categorized as class 3 solvents that have a lower risk to human health and have no negative genotoxicity and long term carcinogenicity [63]. Moreover, ethanol is one of the cleanest among these solvents, considering the renewability and availability as a food-grade solvent, and being cheaper than other solvents [63].

Lin et al. [59] observed that ethyl acetate and ethanol at 2:1 or 1:1 ratios (*v*/*v*) provide a similar yield of lipids (comparison with chloroform/methanol, 2:1, *v*/*v*) from fresh egg yolk, boiled yolk, yolk powder, and raw animal tissues. Probst et al. [109] demonstrated that cyclopentyl methyl ether is an alternative solvent to chloroform and can efficiently extract triacylglycerols from yeast, *Lipomyces starkeyi*. De Jesus et al. [65] tested the traditional methods with green solvents 2-methyl tetrahydrofuran (2-MeTHF) and cyclopentyl methyl ether (CPME) for the extraction of lipids from wet microalgae biomass of *Chlorella pyrenoidosa*. In results, extractions using traditional Bligh and Dyer methods and Folch showed significantly higher yield (113.5–115.1 mg lipids/g biomass), followed by Hara and Radin [16] method (108.66 mg lipids/g biomass). Among the green solvents, the 2-MeTHF/isoamyl alcohol/water system used in the Bligh and Dyer method provided the highest (83.2%) yield of lipids (compared to chloroform/methanol). However, the estimated cost of solvents was a minimum for the Hara and Radin methodology (hexane/isopropanol solvent mixture), costing US$ 167.00/kg of fatty acids. While extraction using the 2-MeTHF/isoamyl alcohol/water system cost 30 times higher (US $4500.00). These observations suggest that based on cost, green solvents are uncompetitive in comparison to fossil-based solvents. However, in the future, the higher production of green solvents may reduce the cost.

### 4.8. Other Methods

Accelerated solvent extraction (ASE; a commercially available pressurized fluid extraction technique) facilitates the rapids and efficient extraction of lipids. Tang et al. [110] recorded the 6.9% higher yield of lipids from dry biomass of *Chlorella vulgaris* utilizing the ASE with chloroform/methanol (2:1, *v*/*v*), compared to conventional extraction using these solvents. The extraction using ASE was highest when extraction temperature of 100 °C, static time of 5 min, a static cycle number of 4 were used. Moreover, with ASE, the solvent consumption and extraction time significantly reduce to nearly 1/2 and 1/10, respectively, without compromising the quality and quantity of extracted lipids [80]. Chen et al. [80] achieved efficient extraction of lipids (in terms of quality and quantity) from dry microalgal biomass (*Scenedesmus*, *Chlorella*, and *Isochrysis* sp.) utilizing the ASE with one cycle of methanol/dimethyl sulfoxide (DMSO) (9:1, *v*/*v*) and two cycles of n-hexane/diethyl ether (1:1, *v*/*v*) extractions performed for 3 min at 125 °C using a 5 mL extraction cell containing 20–50 mg of dry biomass. The advantages and disadvantages of various lipid extraction methods are illustrated in the Table 5.

## 5. Conclusions and Prospects

The chloroform/methanol-based classical extraction methods (Folch method and Bligh and Dyer method) developed more than 60 years ago to extract lipids from animal tissues containing <2% lipids are still used widely, as it provides the high yield of lipids from a wide range of plants and animals samples. Several modifications have been suggested for these methods, for instance, to replace the use of toxic chloroform/methanol, n-hexane/isopropanol (3:2 *v*/*v*) based extraction method suggested by Hara and Radins [16], and isopropanol/cyclohexane/water (8:10:11 *v*/*v*/*v*)-based method developed by Smedes [15] are widely used followed for lipid extraction. Moreover, acidification of extraction solvents by adding 0.5% 6M HCl has been suggested by Retra et al. [17] to the second chloroform wash to increase the recovery of acidic phospholipids. Matyash al [12] developed a high throughput method that uses methyl-tert-butyl ether (MTBE) is followed mainly for animal lipidomics studies. Similarly, An extraction method developed by Welti et al. [14] is extensively used for plant lipidomics, which incorporates a hot isopropanol treatment to inhibit the activity of lipolytic enzymes present in plants.

Soxhlet extraction is also commonly used to extract the crude lipids from dehydrated biomass efficiently. However, continuous heating at the boiling temperature could lead to lipid oxidation and degradation of health liable compounds. In recent years, with the advancement of sorbents materials, solid-phase extraction (SPE) offers high throughput isolation and purification of selected lipids, along with the enrichment of minor lipid classes.

In view of the health and environmental concerns, the use of green solvents, such as supercritical CO_2_, plant-derived terpenes (e.g., D-limonene, p-cymene, and α-pinene), ionic liquids (non-aqueous salt solution) are emerging. The use of green solvents for lipid extraction has been successfully evaluated in yeast and microalgae, and their applicability for extraction lipids from plant and animal tissues needs to be studied. Green solvents are not cost-effective compared to comparison to fossil-based solvents. However, in the future, with the higher demand and production, the cost may be reduced.

Given the increasing use of high-throughput lipidomic analysis, future research should focus on the development of automated workflows for the extraction of lipids from a wide range of samples. This will help increase the efficiency, quality, and reproducibility of the analysis compared to manual methods. Further, future research should evaluate the feasibility of using green solvents for routine lipidomic analysis by comparing extraction efficiency with traditional lipid extraction methods.

## Figures and Tables

**Table 1:** The advantages and disadvantages of major pretreatment methods applied to the efficient extraction of lipids.

Pretreatment Methods	Mode of Action	Advantages	Disadvantages	References
Acid-catalyzed hot-water	Release of bound lipids by uncoupling the lipid-protein and lipid-starch and intermolecular forces	Cost-effective Can be applied for wet biomass High yield of bound lipids	Degradation of thermolabile and acid-sensitive compounds	[34]
Bead beating	Mechanical compaction and shear stress	Cost-effective Continuous module of operation High disruption efficiency Mild operating temperature Suitable for lab-scale to industrial scale	High energy demand Low recovery of lipids from cells with rigid cell wall	[35,36]
Enzyme	Specific enzyme-substrateinteraction	Simple Mild temprtaure conditions No sophisticated instrument required low energy requirements Selective extraction	Long processing time and High cost of enzymes Extraction efficiency depends on the cell wall characteristics	[18,28,29,30,31,32,37]
Expeller press	Mechanical compaction and shear stress	Cost-effective and simple process Solvent-free extraction possible Microwave heating before expeller press can improve the lipid yield	High energy demand Not effective for samples of high moisture content Low recovery of lipids	[38,39,40,41]
High-pressure homogenization (HPH)	Cavitation and shear stress	Simple continuous operating system Can be applied for wet biomass Low solvent requirement Low-temperature extraction Applicable to large-scale	High capital and maintenance cost Less efficient for filamentous microorganisms Undesirable for heat-liable compounds Induced the formation of free fatty acids	[42,43]
High-speed shearing homogenization	Cavitation and shear forces	Suitable for wet and dry biomass Efficient extraction Rapid	Extensive heat generation High energy consumption	[18,44]
Hydrodynamic cavitation	Shear forces, creation, and extinction of cavities	High extraction efficiency from microalgae	High energy consumption Excess heat generation Cavitation reactor designs are at an initial stage Need optimization of critical parameters (orifice plate, inlet pressure, flow rate, cavitation number etc.)	[23,45]
Microwave Irradiation	Temperature increase, molecular energy increase	Makes membranes porous which facilitates the effective extraction of lipid Short operating time More efficient than conventional heating	High energy demand Not suitable for commercial scale High extraction temperature Generation of free radicals	[39,41,46]
Osmotic shock	osmotic pressure-induced cell disruption and the release of the intracellular lipids	Lower energy consumption Easier scale-up High yield	Generation of waste saltwater Time-consuming	[25,47]
Pulsed Electric Field (PEF)	Transient permeabilization of cell membranes	High energetic efficiency Rapid Nonthermal method	High initial capital investment-temperature extraction	[48,49,50,51]
Ultrasonication	Cavitation, acoustic streaming, and liquid shear stress	Extensively used pretreatment method Rapid High yield Energy-efficient process for optimum cell disintegration	Generation of free radicals after prolonged treatment Not investigated for large scale applications	[52,53,54,55,56]

**Table 2:** The list of solvents tested for the efficient extraction of lipids plants, animals, and microbes.

Sample	Solvent Tested	Most Efficient Solvents *	Reference
Argan (*Argania spinosa* L.) seeds	n-Hexane, ethyl acetate, acetone, n-hexane/acetone (1:1, *v*/*v*), ethanol/water (96:4, *v*/*v*), and water	n-Hexane/acetone (1:1, *v*/*v*)	[58]
Fresh egg yolk, boiled yolk, and yolk powder	Ethyl acetate/ethanol (in different ratios) and chloroform/methanol (2:1, *v*/*v*)	Ethyl acetate/ethanol at 2:1 and 1:1 ratios (*v*/*v*)	[59]
Human plasma	1-Butanol/methanol (1:1 and 3:1, *v*/*v*) and chloroform/methanol (2:1, *v*/*v*)	1-Butanol/methanol (1:1, *v*/*v*)	[13]
Krill meal	Acetone, ethanol, isopropanol, ethyl acetate, isohexane, n-hexane, and subcritical butane	Ethanol and isopropanol	[60]
Legumes	Chloroform/methanol (Folch method), n-hexane/isopropanol and n-hexane/acetone	Chloroform/methanol	[61]
Milk	Butanol/methanol (3:1 and 1:1, *v*/*v*), butanol/methanol/chloroform, 3:5:4 *v*/*v*), and chloroform/methanol (2:1, *v*/*v*; Folch method)	Butanol/methanol/chloroform (3:5:4, *v*/*v*)	[62]
Microalga *Tetraselmis* sp. M8	Chloroform/methanol (1:2, *v*/*v*), dichloromethane/methanol (2:1, *v*/*v*), isopropanol/n-hexane (1:1.25, *v*/*v*)	Dichloromethane/methanol (2:1, *v*/*v*)	[26]
Spent coffee grounds	Ethyl acetate, ethanol, isopropanol, and n-propanol	Ethanol	[63]
Thraustochytrids	Chloroform, diethyl ether, ethanol, heptane, n-hexane, isopropanol, methylene chloride, methanol, toluene, and in two solvent combinations at ratios of 1:1, 1:2, and 2:1 (*v*/*v*)	Chloroform/methanol (2:1, *v*/*v*)	[25]

* In terms of extraction yield and health and environmental impact.

**Table 3:** The optimized parameters of supercritical CO_2_ extraction of lipids.

Sample	Optimized Parameters	Reference
Argan seeds	The pressure of 297.71 bar and a temperature of 44.63 °C	[82]
Argan seeds	The pressure of 400 bar and temperature 45 °C	[83]
Grape seeds	The pressure of 500 bar and a temperature of 50 °C, and solvent flow of 8 g/min	[84]
Microalage (20% water)	The pressure of 30 MPa, the temperature of 60 °C, with 0.4 kg/h of CO_2_ and 5% of co-solvent (ethanol)	[81]
Microalga *Tetraselmis* sp. M8	Initial soaking period of 12 h (150 bar, 40 °C), flushing cycle (5 mL/min Flow rate, 30 min)	[26]
Oats (*Avena sativa* L.)	The pressure of 550 bar, the temperature of 47.7 °C, and large particle size (>250 μm)	[85]
Soybean seeds	Extraction with CO_2_/dimethyl ether (DME; 14:1, *v*/*v*) at 20 MPa, 40–60 °C	[86]

**Table 4:** Solid-phase extraction (SFE) of lipid classes.

Sample	Desired Lipid Class	Sorbent	Separation Principle	Reference
Clam (*Corbicula fluminea*)	Phospholipids	Titania-coated fibrous silica (TiO2/KCC-1)	Hydrophilic interaction	[103]
Extra virgin olive oil	Phospholipids	Weak anionic exchange phase containing charged piperazine units, or graphitized carbon black	Ionic and lipophilic interactions	[104]
French fries	Monounsaturated fatty acid methyl esters	Silver (Ag) nanoparticles-coated monolithic	Ag^+^-like affinity interaction	[105]
Human breast milk	Phospholipids and glycerolipids	Mixture of C_18_ and zirconia-coated silica gel	Hydrophobic and Lewis acid/base interaction	[106]
*Hypophthalmichthys nobilis*	Phospholipids	Sulfobetaine (3-(trimethylammonio)propane-1-sulfonate)	Zwitterionic hydrophilic interaction	[107]
Milk powder-based products	Oxysterols	C_18_ silica	Hydrophilic interaction	[108]

**Table 5:** Advantages and disadvantages of various lipid extraction methods.

Extraction Method	Advantages	Disadvantages	References
Accelerated solvent extraction (ASE)	Automated and rapid (≈ 1 min) extraction method Low consumption of solvents commercially available technique	High extraction temperature Special ASE Instrument required	[80,110]
Green solvent assisted extraction	Environment-friendly, non-toxic Food quality grade product	Required an additional demulsification step	[31,63,73,111,112]
Maceration and solvent extraction	Standard methods for extraction High yield of lipids	Laborious multistep process Use of toxic solvents Solvent residues in the product	[113,114,115]
Soxhlet extraction	Standard method of lipid extraction High yield of lipids	Time-consuming Use of toxic solvents High extraction temperature	[77,78,79]
Supercritical CO_2_	Environment-friendly, non-toxic, and non-flammable (CO_2_) Solvent-free extraction Food quality grade product Minimum/zero post-extraction processing	High instrumentation cost High energy requirements Low yield of polar lipids	[81,82,84,85]

## Data Availability

Data contains within the article.
